# Plastome phylogenomics and historical biogeography of aquatic plant genus *Hydrocharis* (Hydrocharitaceae)

**DOI:** 10.1186/s12870-022-03483-2

**Published:** 2022-03-08

**Authors:** Zhi-Zhong Li, Samuli Lehtonen, Andrew W. Gichira, Karina Martins, Andrey Efremov, Qing-Feng Wang, Jin-Ming Chen

**Affiliations:** 1 Key Laboratory of Aquatic Botany and Watershed Ecology, Wuhan Botanical Garden, Chinese Academy of Sciences, Wuhan, 430074 China; 2 Center of Conservation Biology, Core Botanical Gardens, Chinese Academy of Sciences, Wuhan, 430074 China; 3 Herbarium, Biodiversity Unit, University of Turku, 20014 Turku, Finland; 4 Sino-Africa Joint Research Center, Chinese Academy of Sciences, Wuhan, 430074 China; 5 Departamento de Biologia, Universidade Federal de São Carlos, Sorocaba, 18052-780 Brazil; 6 Research Center of Fundamental and Applied Problems of Bioecology and Biotechnology of Ulyanovsk State Pedagogical University, 4/5, Lenin Square, 432071 Ulyanovsk, Russia

**Keywords:** *Limnobium*, Plastome, Divergence time, *Hydrocharis chevalieri*

## Abstract

**Background:**

*Hydrocharis* L. and *Limnobium* Rich. are small aquatic genera, including three and two species, respectively. The taxonomic status, phylogenetic relationships and biogeographical history of these genera have remained unclear, owing to the lack of Central African endemic *H. chevalieri* from all previous studies. We sequenced and assembled plastomes of all three *Hydrocharis* species and *Limnobium laevigatum* to explore the phylogenetic and biogeographical history of these aquatic plants.

**Results:**

All four newly generated plastomes were conserved in genome structure, gene content, and gene order. However, they differed in size, the number of repeat sequences, and inverted repeat borders. Our phylogenomic analyses recovered non-monophyletic *Hydrocharis*. The African species *H. chevalieri* was fully supported as sister to the rest of the species, and *L. laevigatum* was nested in *Hydrocharis* as a sister to *H*. *dubia*. *Hydrocharis-Limnobium* initially diverged from the remaining genera at ca. 53.3 Ma, then began to diversify at ca. 30.9 Ma. The biogeographic analysis suggested that *Hydrocharis* probably originated in Europe and Central Africa.

**Conclusion:**

Based on the phylogenetic results, morphological similarity and small size of the genera, the most reasonable taxonomic solution to the non-monophyly of *Hydrocharis* is to treat *Limnobium* as its synonym. The African endemic *H. chevalieri* is fully supported as a sister to the remaining species. *Hydrocharis* mainly diversified in the Miocene, during which rapid climate change may have contributed to the speciation and extinctions. The American species of former *Limnobium* probably dispersed to America through the Bering Land Bridge during the Miocene.

**Supplementary Information:**

The online version contains supplementary material available at 10.1186/s12870-022-03483-2.

## Backgroud

*Hydrocharis* L., an aquatic monocot genus, has a broad native distribution in tropical and temperate regions of the Old World [1]. As traditionally circumscribed, the genus comprises only three allopatric species: *Hydrocharis chevalieri* (De Wild.) Dandy, *H. morsus-ranae* L. and *H. dubia* (Blume) Backer. Among them, *H. chevalieri* is only found in small sedge swamps or wetlands in central Africa [1, 2]. It is easily distinguished from the other two species by unique morphological features, e.g., long and erect petioles and the number of primary veins (> 4) in leaf lamina [1]. In comparison, *H. morsus-ranae* and *H. dubia* are more widely distributed. The native range of the former covers West and North Eurasia, whereas the latter is natively distributed in Southeast Asia [1, 3]. Beyond their natural range, *H. morsus-ranae* and *H. dubia* have invaded Northeast America and Australia, respectively [3], and cause serious damage to the local environment [2]. However, due to the deterioration of the aquatic habitats resulting from human activities, *H. morsus-ranae* and *H. dubia* have been considered threatened in part of their native distributions [3]. *Hydrocharis* and the closely related *Limnobium* are unusual in Hydrocharitaceae by having aerial leaves only, and therefore are important for understanding the evolution of aquatic adaptations in the family [1, 4]. Additionally, *H. morsus-ranae* has been shown to have the ability to accumulate heavy metal elements making it a possible bioindicator of tracing element pollution in freshwaters [5].

Since the taxonomic revision of the genus *Hydrocharis* by Cook and Lüönd [1], only a few studies have focused on the systematics and taxonomy of the genus. Chen et al. [4] reappraised the generic relationships of Hydrocharitaceae using eight genes representing nuclear, plastid, and mitochondria genomes. The genus *Hydrocharis* was resolved as monophyletic and highly supported sister of *Limnobium*, a genus with two currently accepted species [6]. In that study, both *Limnobium* species were sampled, but one of the *Hydrocharis* species remained unsampled. Similarly, Ross et al. [7] resolved *Hydrocharis* and *Limnobium* as sisters in a phylogenomic analysis of 83 plastid genes, but they only sampled one species of each genus. Bernardini and Lucchese [8] further studied the phylogeny of Hydrocharitaceae using a dataset of ITS and five plastid genes. In their study, the taxon sampling and results concerning *Limnobium* and *Hydrocharis* were comparable with Chen et al. [4]. They suggested that these two genera could be merged into a single genus. This has also been supported by morphologic evidence [6, 9, 10] and recently Plant Gateway [11] concluded – without further justification – that *Limnobium* is part of *Hydrocharis*. However, the systematic relationships of *Hydrocharis* and *Limnobium* remain unclear due to the lack of morphologically intermediate Central African endemic *H. chevalieri* from all previous studies.

Previous biogeographic analyses suggested that the most recent common ancestor (MRCA) of the *Hydrocharis-Limnobium* clade originated in the Oriental region approximately 15.9 Ma, and long distance dispersal (LDD) might contribute to its current distributions [4]. However, numerous fossils, including more than 10 *Hydrocharis* species, which mainly occurred during the Oligocene and Miocene epochs, were discovered in Europe and the Far East [3, 12–15]. No fossils have been found in Africa. The biogeographic history of the African species *H. chevalieri* has been unsolved, limiting a comprehensive understanding of the biogeographic history of the genus.

As the plastome has become more easily retrievable, it has dramatically enhanced the resolution of phylogenetic relationships at various taxonomic levels [7, 16–18]. Here, we sequenced and assembled plastomes of all three *Hydrocharis* species and *Limnobium laevigatum*. The objectives of this study were to 1) investigate the plastome evolution in this group; 2) clarify the phylogenetic relationships of *Hydrocharis* and *Limnobium*; and 3) infer the historical biogeography of the group.

## Results

### Plastome feature

**Fig. 1:**
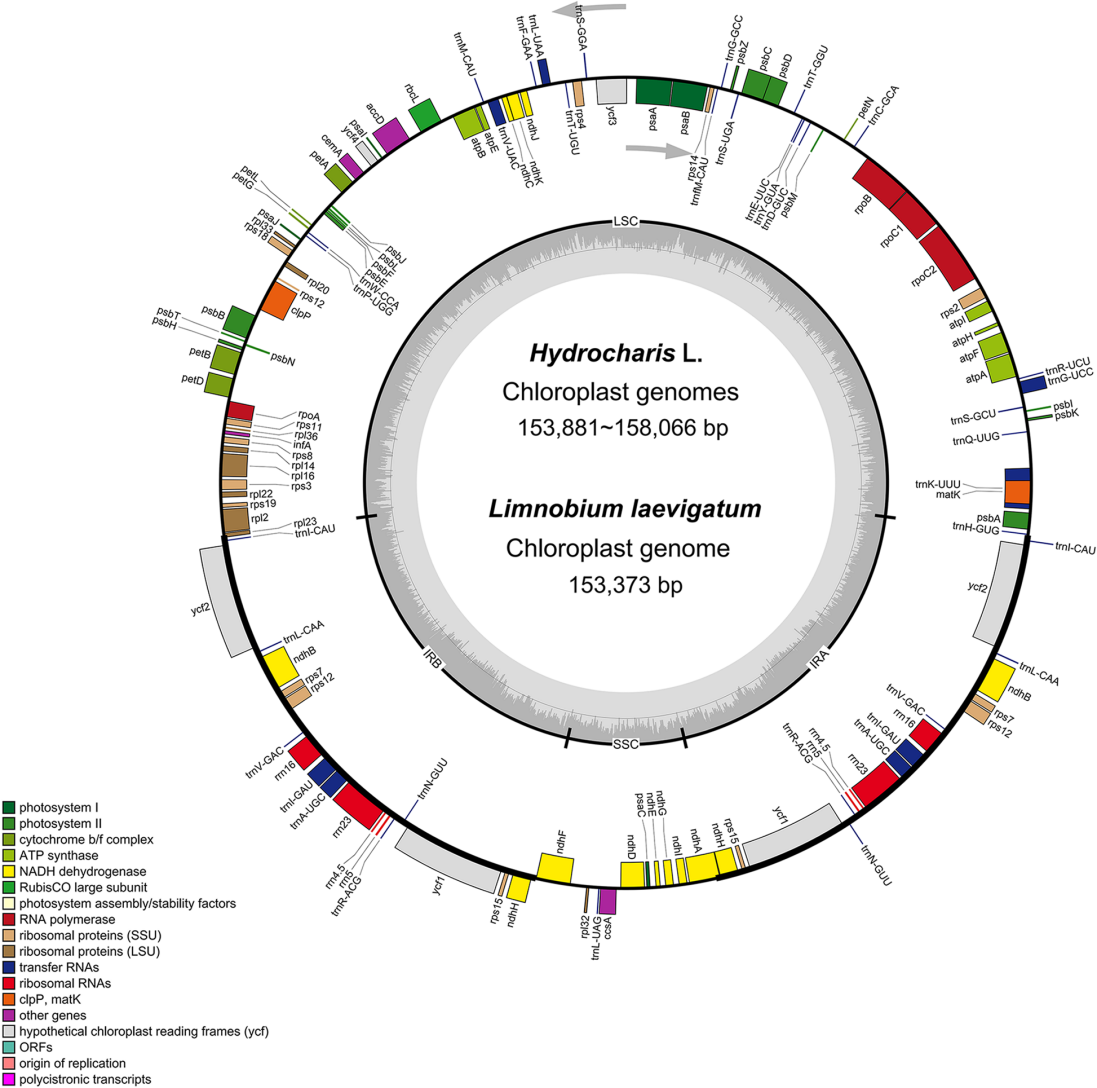
Plastome maps of *Hydrocharis* species and *Limnobium laevigatum*. The arrowhead shows the inverted region

After de novo assembly, we obtained the complete plastome of three *Hydrocharis *species and *L. laevigatum* from genome skimming data. The coverage of each plastome ranged from ~ 807× to ~ 878× (Table 1). The size of newly assembled plastomes ranged from 153,373 bp to 159,698 bp and exhibited the typical quadripartite structure (Fig. 1), including a pair of IRs (22,292–30,605 bp) separated by the Large Single Copy (LSC, 85,578–89,581 bp) and Small Single Copy (SSC, 11,278–21,476 bp) regions (Table 1). Except for *rps16*, lost from the plastome of *H. chevalieri*, the other plastomes encoded 113 unique genes, which is similar to most of the plastomes in angiosperms. Unique genes comprised 79 PCGs, 30 tRNA genes, and four rRNA genes. Both the average gene density (~ 0.8) and the overall GC content (~ 37%) of plastomes were conserved across the species. Furthermore, a small inversion of the *trnN-GUU* gene was detected in *H. dubia* plastome (Fig. 1).

**Table 1:** Detailed information of the newly assembled plastomes

Name of organism	*Hydrocharis dubia*	*Hydrocharis chevalieri*	*Hydrocharis morsus-ranae*	*Limnobium laevigatum*
Voucher No.	HIB-LZZ-SB12	HIB-LZZ-Cam19	14,513	HIB-CJM-Bra50
Collection Sites	China; Wuhan	Cameroon; Mangueme	–	Brazil; Rio de Janeiro
GenBank accession number	OK326868	OK326871	OK326869	OK326870
SRA accession number	SRR16469680	SRR16469681	SRR16469679	SRR16469678
Raw data (G)	8.5	7.7	8.5	7.4
Clean data (G)	8.2	7.5	8.1	7.2
Mean coverage	828×	807×	872×	878×
Genome size (bp)	159,698	158,066	153,881	153,373
LSC length (bp)	89,581	85,578	88,098	87,313
SSC length (bp)	18,211	11,278	20,695	21,476
IR length (bp)	25,953	30,605	22,544	22,292
Number of genes	113	112	113	113
Number of protein-coding genes (duplicated in IR)	79 (4)	78 (6)	79 (3)	79 (3)
Number of tRNA genes (duplicated in IR)	30 (7)	30 (7)	30 (7)	30 (7)
Number of rRNA genes (duplicated in IR)	4 (4)	4 (4)	4 (4)	4 (4)
Number of genes with one intron (two introns)	16 (2)	15 (2)	16 (2)	16 (2)
Proportion of coding to non-coding regions	0.73	0.74	0.72	0.73
Average gene density (genes/kb)	0.8	0.82	0.83	0.83
GC content (%)	37.2	37	37.2	37

### Comparison of border regions and sequence identity

**Fig. 2:**
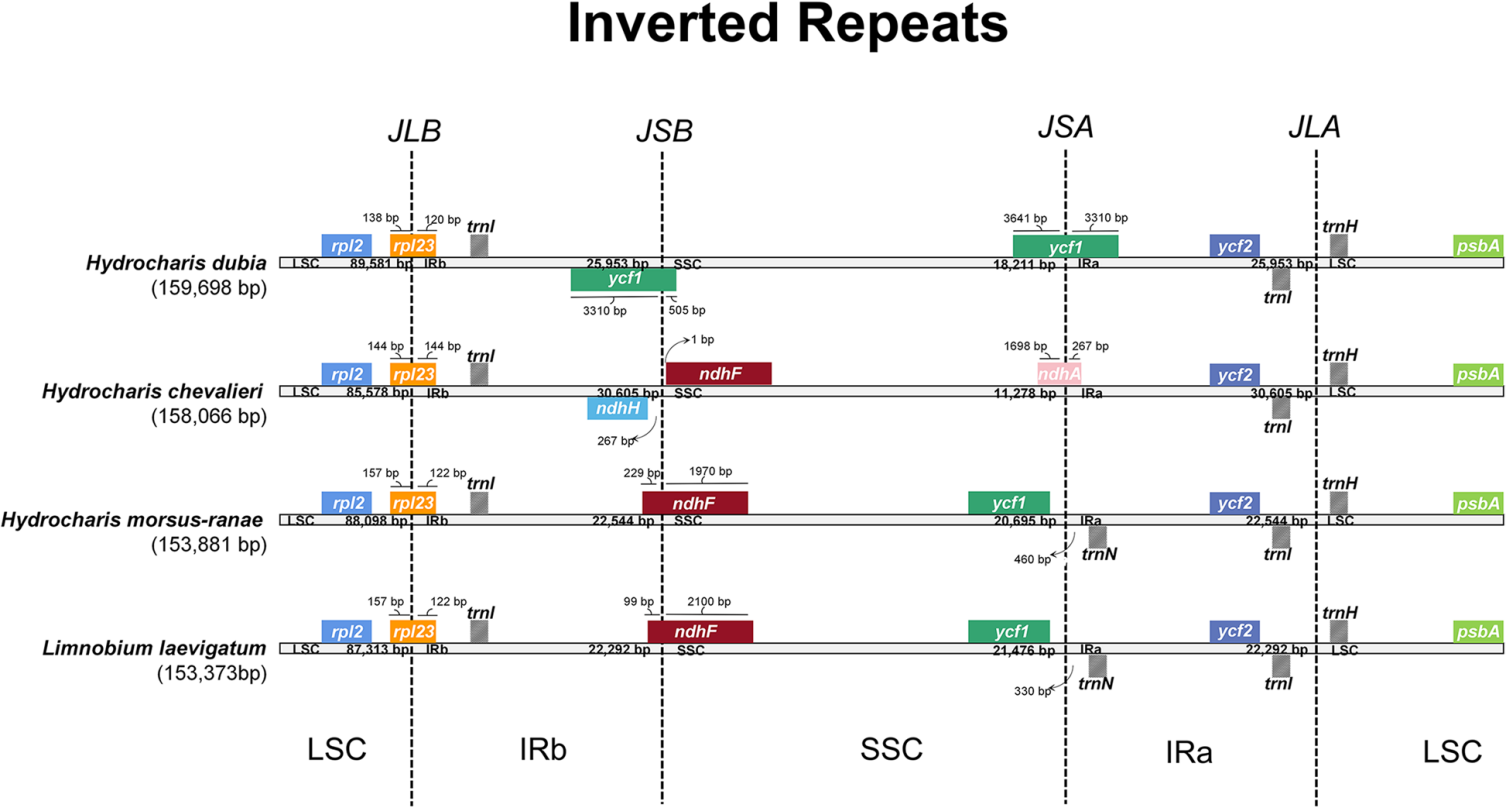
Comparison of the boundary of *Hydrocharis* species and *Limnobium laevigatum*

The IR/LSC and IR/SSC junctions were compared to assess the IR expansion or contraction of the plastome. Here, the LSC/IRa (JLA) and LSC/IRb (JLB) junctions were identical for all four species; IR expanded into the *rpl23* gene at the JLB, and the JLA were located at the *trnI*-*trnH* intergenic spacer (Fig. 2). substantial divergence was detected at the SSC/IRa (JSA) and SSC/IRb (JSB) junctions*.* In *H. dubia* IR expanded into the *ycf1* gene at the JSA, whereas in *H. chevalieri* the *ndhA* gene overlapped with the JSA and in *H. morsus-ranae* and *L. laevigatum* JSA was placed in *ycf1-trnN* intergenic spacer. In comparison, at the JSB the IR regions expanded into genes in *H. dubia* (*ycf1*), *H. morsus-ranae* (*ndhF*) and *L. laevigatum* (*ndhF*). The JSB of *H. chevalieri* lay in the *ndhH-ndhF* intergenic spacer.

Both the sequence identity analysis and Pi values revealed a high differentiation among the newly assembled plastomes. The divergent regions were mostly derived from IGS regions (Fig. S1,S2), e.g., *ycf1*-*trnN*-*GUU*, *rps16*-*trnQ*-*UUG*, *trnI*-*CAU*-*ycf2*, *ycf2*-*trnI*-*CAU*, *trnK*-*UUU*-*rps16*. Additionally, the top five PCGs, *ycf1*, *rps12*, *rps18*, *rps3*, *accD,* showed relatively high nucleotide diversity and had the potential to be developed into molecular markers for further phylogenetic research.

### Sequence repeat and SSR analysis

**Fig. 3:**
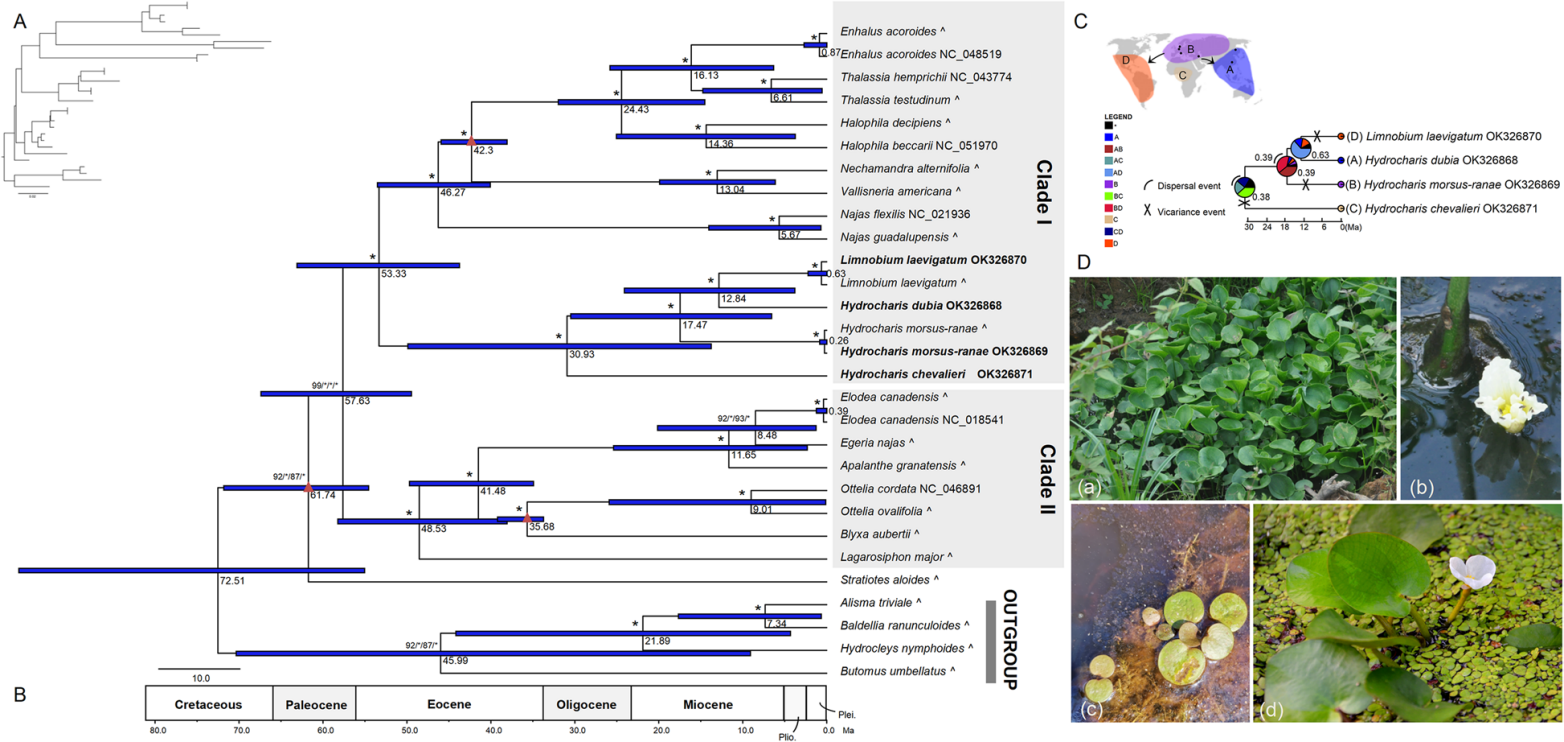
Phylogenomic and biogeographic analysis of *Hydrocharis*. **A** the phylogenetic tree of Hydrocharitaceae was reconstructed from 78 PCGs sequences. **B** the dated phylogenetic tree of Hydrocharitaceae. Caret indicates the data downloaded from Ross et al. (2016). Asterisks represent bootstrap support = 100/posterior probability = 1.00; the first two values represent unpartitioned data, and the last two values represent partitioned data. Triangles indicate the fossil calibration nodes and numbers below branches refer to the mean divergent time estimates. Error bars indicate 95% highest posterior distributions. **C** the biogeographic analysis of the genus *Hydrocharis*. The black points indicates the location of *Hydrocharis* fossils [3]. **D** Plant photos of *Hydrocharis* and *Limnobium* were taken by Zhi-Zhong Li. a-b, *H. chevalieri*; c, *L. laevigatum*; and d, *H. dubia*

The SSR analysis revealed a similar number of SSRs (Table S1) , ranged from 64 (*H. morsus-ranae*) to 69 (*H. dubia*), within *Hydrocharis* species. In contrast, a clearly higher number of SSRs was detected in *L. laevigatum* (85). Moreover, species of *Hydrocharis* had the largest number of tri-, tetra-, and hexa-nucleotide repeats, whereas *L. laevigatum* was rich in mono-, and di- nucleotide repeats were more common in *L. laevigatum* (Table S1). Four types of repeat elements, forward, palindromic, reverse, and complementary, were identified for *Hydrocharis* and *L. laevigatum* plastomes. The total number varied from 383 (*H. morsus-ranae*) to 689 (*H. dubia*) repeats of 30–317 bp length for the newly generated plastomes (Fig. S3). Forward repeats were the most common and complementary repeats were the rarest repeat type. Based on the repeat size, we further divided all repeats into the following categories: 35–45 bp, 46–60 bp, 61–75 bp, 76–90 bp, and > 90 bp (Fig. 3). At least half of the repeats belonged to the size class of 35–45 bp, and the majority of long repeats (> 90 bp) were located in the plastome of *H. dubia* (107 repeats).

### Phylogenetic analysis

Both ML and BI methods, regardless of partitioning strategy, revealed completely identical topologies and robust support for most of the nodes (Fig. 3). Two major clades were identified with high support (BS = 99/100, PP = 1/1) and the genus *Stratiotes* was fully resolved as the sister to these two clades. *Hydrocharis* and *Limnobium* were clustered with full support (BS = 100/100, PP = 1/1) and resolved as the sister group of the remaining genera within Clade I. However, *Hydrocharis* did not form a monophyletic group, but *L. laevigatum* was nested within it as a fully supported sister to *H. dubia*. Additionally, African species *H. chevalieri* was fully supported as sister to the rest of the species within (*Hydrocharis* + *Limnobium*) group, and *H. morsus-ranae* shared a common ancestor with (*L. laevigatum* + *H. dubia*).

### Divergence time estimation and biogeographical reconstruction

The MRCA of Hydrocharitaceae was inferred at ca. 61.7 Ma [Fig. 3, 95% highest posterior densities (HPD): 54.6–71.8 Ma] and the Clade I and Clade II split from each other at 57.6 Ma (95% HPD: 49.5–67.4 Ma). In Clade I, *Hydrocharis-Limnobium* initially diverged from the remaining genera at ca. 53.3 Ma (95% HPD: 43.7–63.1 Ma), and then began to diversify at ca. 30.9 Ma (HPD: 13.8–49.9 Ma). The MRCA of *H. dubia* and *L. laevigatum* was dated at ca. 12.84 Ma (95% HPD: 3.8–24.1 Ma) and diverged of *H. morsus-ranae* and the remaining species at ca. 17.5 Ma (95% HPD: 6.6–30.5 Ma).

Biogeographical reconstruction was implemented using RASP [19] with DIVALIKE model supported as the best-fit model (LnL = − 8.23, AICc = 24.46, AICc_wt = 0.56; Table 2) from BioGeoBEARS analysis. However, the ancestral area remained uncertain. The scenario with the highest probability (39%) suggested that the genus originated in Europe and Central Africa, followed by diversification due to three vicariance and two dispersal events (Fig. 3).

**Table 2:** Results of BioGEoBEARS models for the ancestral area reconstruction

Model	LnL	num params	d	e	j	AICc	AICc_wt
DEC	−9.26	2	0.018	0.027	0	26.52	0.2
DEC + J	−6.02	3	1.00E-12	1.00E-12	0.19	30.03	0.035
**DIVALIKE**	**−8.23**	**2**	**0.008**	**3.60E-05**	**0**	**24.46**	**0.56**
DIVALIKE+J	−5.28	3	1.00E-12	1.00E-12	0.15	28.56	0.072
BAYAREALIKE	−9.82	2	0.027	0.047	0	27.65	0.11
BAYAREALIKE+J	−6.66	3	1.00E-07	1.00E-07	0.2	31.32	0.018

## Discussion

### Plastome evolution

Similar to the most reported plastomes of angiosperms [17, 20], all the plastomes in our study exhibited relatively conservative genome structure, gene content, and gene order [21, 22]. However, when compared to *H. morsus-ranae* and *L. laevigatum*, *H. dubia* (159,698 bp) and *H. chevalieri* (158,066 bp) had larger plastomes. One reason for plastome size variation is the expansion or contraction of IRs [23, 24]. Indeed, *H. dubia* and *H. chevalieri* had one (*ycf1*) and three more genes (*ycf1*, *rps15*, and *ndhH*) in IRa, respectively, than the other two species. In addition, typical patterns of the plastome evolution associated with the fluctuation of plastome length include the gain/loss of genes, pseudogenization, and variations in intergenic regions [24–26]. Our results revealed the loss of gene *rps16* in African endemic *H. chevalieri*. This gene has been reported missing from plastomes in many plant groups, e.g., Oxalidaceae [27, 28], Podostemaceae [29], and Violaceae [25]. It seems that *rps16* has been frequently transferred to the nuclear genome [20, 30]. Additionally, a small inversion of the *trnN-GUU* gene near the IR boundary was detected in *H. dubia*. The inversion was likely related to the shift in the IR boundary (Fig. 2), as identified and demonstrated by Zhu et al. [31].

Previous studies [4, 8] have applied only five plastid genes (*rbcL*, *matK*, *rpoB*, *rpoC1*, *trnK*) to resolve the phylogeny with the genus *Hydrocharis*. However, there was only low nucleotide diversity(Pi < 0.06) within these five genes, which might have affected the accuracy and resolution of phylogenetic reconstruction [32]. In our study, a number of regions (Fig. S2), including five plastid genes (*ycf1*, *rps12*, *rps18*, *rps3*, *accD*) and five intergenic spacers (*ycf1*-*trnN*-*GUU*, *rps16*-*trnQ*-*UUG*, *trnI*-*CAU*-*ycf2*, *ycf2*-*trnI*-*CAU*, *trnK*-*UUU*-*rps16*)*,* were detected as hotpots and may be helpful even in population genetic studies in the genus.

The high frequency of repeat sequences has been demonstrated to be one of the leading causes of plastome rearrangement and divergence [33]. Our analysis identified many repetitions (> 350) with more than 30 bp length in all newly assembled plastomes. Among these, short repeat sequences (30–45 bp) were dominant, similar to other plant plastomes that have not undergone large-scale structural variation. SSRs are widely distributed in plant plastomes and exhibit relatively high polymorphism, which can be used in population genetics [34–36]. The majority of SSRs were mono- and di- nucleotides in four newly assembled plastomes, which have been reported in other angiosperm plastomes, e.g., *Primula* [37], *Dendrosenecio* [18], and *Oxalis* [28]. The cpSSRs reported here could be used as genetic markers for future studies into the genetic diversity of *Hydrocharis*.

### Phylogeny and biogeographical reconstruction

Prior to this study, no molecular information was available for the African endemic *H. chevalieri*. Here we have clarified the relationships within *Hydrocharis* by assembling and reporting the plastomes of all three *Hydrocharis* species and *Limnobium laevigatum*. Unlike previous studies [4, 8], *H. morsus-ranae* was fully supported (Fig. 3, BS = 100/100; PP = 1/1) as sister to the (*L. laevigatum* + *H. dubia*), instead of getting together with *H. dubia*, which was likely due to the inadequacy of *L. spongia* in our current study. Moreover, we recovered non-monophyletic *Hydrocharis*. The genus *Limnobium* represented here by *L. laevigatum* was nested in *Hydrocharis* as sister to *H. dubia* with robust support (Fig. 3, BS = 100/100; PP = 1/1). This contradicting result might be because previous studies did not include *H. chevalieri* and used only a limited number of molecular markers [4, 8]. Furthermore, a series of morphological features support the current phylogenomic relationships (Fig. 3). Vegetatively, *Hydrocharis* and *Limnobium* are indistinguishable, but *H. chevalieri* has stout, erect petioles, and laminas with a large number of primary veins, some of which originate from the lower half of the midrib [6]. The remaining species have slender petioles and primary veins originating from the point of petiolar attachment [6]. *Limnobium* differs from *Hydrocharis* by its rudimentary petals [6]. Given the morphological similarity and small size of the genera, only two species in *Limnobium* and three species in *Hydrocharis*, the most reasonable taxonomic solution to the non-monophyly of *Hydrocharis* is to treat *Limnobium* as the synonym of *Hydrocharis*. Nomenclatural combinations already exist, i.e., *Hydrocharis spongia* Bosc and *Hydrocharis laevigata* (Willd.) Byng & Christenh.

Our time-calibrated tree indicated stem node age of about 53 Ma for *Hydrocharis* (Fig. 3), which is similar to the previously reported median age of 54.7 Ma [4] and in agreement with the oldest known *Hydrocharis* fossils from the Eocene [38]. The crown age of *Hydrocharis* was estimated to be approximately 31 Ma, much older than the 15.9 Ma reported in Chen et al. [4] based on analysis that lacked *H. chevalieri*, the sister species of the remaining genus. Based on our results, *Hydrocharis* mainly diversified in the Miocene, which is consistent with the time inferred for many other extant plant species [39, 40]. Rapid climate change during the Miocene may have contributed to the speciation of *Hydrocharis* as well as extinctions, given that the fossil diversity is high in comparison to the limited extant diversity [41, 42]. Additionally, paleoclimatic changes may have induced such changes in water bodies that some groups were driven to extinction [43]. This might explain why there are only three extant species, and a majority of fossil groups only occurred in Europe during the Miocene [3].

The area of origin remained uncertain for the genus because several alternative areas with low probabilities were recovered for the deeper nodes (Fig. 3). This may, of course, also reflect a widespread ancestral distribution. Although, *L. spongia* from America was not sampled here, our model with the highest probabilities for the ancestor of the genus in Europe, Central Asia, and Central African regions (Fig. 3) contrasted the biogeographical model of Chen et al. [4], which suggested origination in the Oriental area. Additionally, our results indicated that at least three vicariance and two dispersal events had shaped the current distribution of the genus (Fig. 3). One dispersal event was from Central Africa to Europe and Central Asia, probably via the last direct connection between Eurasia and Africa prior to the Miocene. The rapid climate change during the Miocene likely resulted in the isolation of *H. chevalieri*, as well as contributed to the extinction of many *Hydrocharis* species in Europe, as also proposed for *Pyrularia* [44], *Allioideae* [45] and some Compositae groups [46]. And other one dispersal route from Europe and Central Asia to America, East and Southeast Asia. The Bering Land Bridge (BLB) has been proved to play an important role in the dispersal of different plant lineages between Eurasia and America [47–49]. The former genus *Limnobium*, naturally recorded only in America, may have dispersed to America through the BLB during the Miocene [49, 50]. The LDD event might be interpreted by the transport of seeds or stolons in mud on the feet of aquatic birds, and It has been considered a credible explanation for the LDD of aquatic groups [4, 17]. The geographic isolation between Eurasia and America likely contributed to the isolation of *Limnobium*. Although the phylogeny and biogeographic history of *Hydrocharis* has been greatly improved here, it should be mentioned that the incomplete sampling of *Limnobium* in America and only one individual was used for each species. There is still a need to include *L. spongia* and more populations to solve a more detailed biogeographic history of *Hydrocharis*.

## Methods

### Taxon sampling and DNA extraction

All the samples used in this study were listed in Table 1. The fresh leaves of two *Hydrocharis* species, *H. chevalieri* and *H. dubia*, were collected in 2019 from Cameroon and China, respectively. *Limnobium laevigatum* was sampled from a small swamp in Brazil. The field collection followed the ethics and legality of the local government and was permitted by the government. Total genomic DNA of these three species was extracted from silica-dried leaves using the MagicMag Genomic DNA Micro Kit (Sangon Biotech, Shanghai, China) following the manufacturer’s protocol. The genomic DNA of *H. morsus-ranae* was retrieved from the Kew DNA bank (ID: 14513) and used for the subsequent analysis.

### Genome skimming, plastome assembly, and annotation

The construction of all sequencing libraries and genome skimming was carried out following Li et al. [17]. Approximately 8.5G paired-end reads (150 bp) were yielded for each sample. After trimming and filtering using Fastp v. 0.20.0 [51] with default parameters, at least 7G clean reads were obtained. The complete plastomes were assembled de novo using GetOrganelle v. 1.7.5 with the recommended settings [52]. Web applications Geseq [53] and PGA [54] were applied to annotate genes in the newly generated plastomes using default settings, with manual adjustment of the start/stop codons through comparison with a reference plastome (*Ottelia alismoides*; NC_057494). Circular maps of plastome were created using OGDRAW v1.3.1 [55]. All newly generated sequencing data and annotated plastomes were submitted to the GenBank (Table 1).

### Comparative analysis and divergence hotspot identification

The web-based software mVISTA [56] was used to identify the sequence and structural variations in *Hydrocharis* and *Limnobium*, using the plastome of *H. dubia* as reference. The comparison of expansions/contraction of IR in *Hydrocharis* and *Limnobium* was conducted using Geneious v 5.6.3 [57].

All the protein coding genes (PCGs) and intergenic spacers (IGSs) were extracted separately, and aligned in MAFFT v. 7.221 [58] with default settings. The program DnaSP v.6 [59] was used to assess the nucleotide diversity (Pi) for all PCGs and IGS of the studied plastomes. Additionally, the online application MISA [60] was employed to predict the simple sequence repeats (SSRs) for each plastome. The minimum number of repetitions was set to 10, 5, 4, 3, 3, and 3, for mono-, di-, tri-, tetra-, penta-, and hexanucleotides, respectively. Furthermore, we explored the forward, reverse, palindromic, and complementary repeats for each plastome using REPuter web-based tools [61], with a minimum repeat size of 30 bp and Hamming distance equal to 3.

### Phylogenetic analysis

Six plastomes of Hydrocharitaceae (*Elodea canadensis* NC_018541; *Enhalus acoroides* NC_048519; *Halophila beccarii* NC_051970; *Najas flexilis* NC_021936; *Ottelia cordata* NC_046891; *Thalassia hemprichii* NC_043774) were downloaded from GenBank. The PCGs were extracted from each of the plastomes and used in the phylogenetic analysis. A total of 78 PCGs in 15 Hydrocharitaceae species, three Alismataceae species (*Alisma triviale*, *Baldellia ranunculoides*, and *Hydrocleys nymphoides*), and *Butomus umbellatus* representing Butomaceae were retrieved from Ross et al. [7]. Finally, we compiled all 78 PCGs from 29 samples for our phylogenetic analysis (Table S2). Three Alismataceae species and *B. umbellatus* were used as outgroups.

All PCGs were aligned using MAFFT v. 7.221 [58] and ambiguously aligned regions were removed using trimAl v. 1.2 [62]. Unpartitioned and partitioned strategies of both Maximum likelihood (ML) and Bayesian inference (BI) methods were utilized for phylogenetic inference. For the unpartitioned strategy, we concatenated all 78 PCGs sequences as a supermatrix to infer the phylogenetic relationship with the best-fit model of nucleotide substitution estimated by ModelFinder [63]. For partitioned strategy, PartitionFinder 2 [64] was used to select the best-fit partitioning schemes and models for 78 PCGs via the rcluster algorithm. All phylogenetic analyses were performed with ML using IQ-Tree v. 1.6.12 [65] and BI using MrBayes v. 3.2.7 [66]. An ultrafast bootstrap approximation was used to estimate ML branch support values with 1000 replicates. BI was run using two independent runs of 8 million generations, and four Markov chains were set for each run, sampling trees every 5000 generations. After verifying that an average standard deviation of split frequencies was less than 0.001, the initial 25% of the trees were discarded as burn-in. A consensus tree with Bayesian posterior probabilities (PP) was constructed from the remaining trees.

### Molecular dating and ancestral area reconstruction

Three credible fossils from Hydrocharitaceae with Lognormal prior distributions were employed to calibrate the divergence times of the lineages within Hydrocharitaceae, following previous studies [4, 17, 67]. The first calibration point was assigned between *Stratiotes* and the rest of the Hydrocharitaceae based on the fossil seeds of *Stratiotes* from the Paleocene-Eocene boundary [67]. This node was constrained to a minimum of 54.5 Ma (offset = 54.5, mean = 1.0, SD = 1.0). The fossil *Thalassites* was applied to constrain the stem node of *Enhalus* + *Halophila* + *Thalassia* to the Middle Eocene. Thus, the split between (*Enhalus* + *Halophila* + *Thalassia*) and (*Nechamandra* + *Vallisneria*) was constrained to a minimum of 38.0 Ma (offset = 38.0, mean = 0.8, SD = 0.9). The oldest fossil from *Ottelia* recorded in the Upper Eocene was used to constrain the split between *Blyxa* and *Ottelia* with a minimum age of 33.7 Ma (offset = 33.7, mean = 1.1, SD = 1.2). An uncorrelated Lognormal relaxed clock model with Yule tree prior was applied for molecular dating implemented in BEAST v.1.10.4 [68]. We ran 8.0 × 10^8^ iterations of Markov chain Monte Carlo (MCMC) and sampled every 2000 iterations. The program Tracer v. 1.7.1 [69] was used to check the effective sample size (ESS) for the convergence of each parameter discarding the initial 25% generations as burn-in.

According to the existing distribution patterns of *Hydrocharis* [1, 3], four major areas were identified for biogeographic analysis: A) East and Southeast Asia; B) Europe and Central Asia; C) Central Africa; D) America. The ancestral states were reconstructed using the BioGeoBEARS package [70] implemented in RASP v. 4.0 [19]. The best fit biogeographic model was selected by BioGeoBEARS [70] based on the Akaike Information Criterion cumulative weight (AICc_wt). The condensed tree and 100,000 sampled trees from BEAST analysis were used as input.

## Conclusion

In this study, we firstly assembled plastomes of three *Hydrocharis* species and *Limnobium laevigatum.* Our phylogenomic analysis recovered non-monophyletic *Hydrocharis* and fully supported *H. chevalieri* as a sister to the rest of the species within the (*Hydrocharis* + *Limnobium*) group. Based on the morphological and phylogenomic evidence, we suggested treating *Limnobium* as the synonym of *Hydrocharis*. Moreover, we reappraised the divergence time and historical biogeography of *Hydrocharis*. *Hydrocharis* mainly diversified in the Miocene, rapid climate change during the Miocene may have contributed to the speciation of *Hydrocharis* and extinctions. The American members of the genus, i.e. former *Limnobium*, probably have dispersed to America through the Bering Land Bridge during the Miocene. In summary, our study provides new insights into the plastome evolution, phylogeny, and biogeography of the genus *Hydrocharis*.

## Supplementary Information

**Additional file 1: Figure S1.** Genome alignment of plastomes of *Hydrocharis* species and *Limnobium laevigatum. H. dubia* was used as a reference.

**Additional file 2: Figure S2.** Nucleotide diversity hotspot regions in plastomes of *Hydrocharis* species and *Limnobium laevigatum.*

**Additional file 3: Figure S3.** Analysis of repeat sequences in plastomes of *Hydrocharis* species and *Limnobium laevigatum*. (A) Total number of four repeat types. (B) Number of repeats divided by size.

**Additional file 4: Table S1.** Distribution of SSRs in plastomes of *Hydrocharis* species and *Limnobium laevigatum.*

**Additional file 5: Table S2.** List of the species downloaded from GenBank and Ross et al. (2016) used for phylogenomic and dating analyses in this study.

## Data Availability

All newly annotated plastomes in this study are available from the National Center for Biotechnology Information (NCBI) (accession numbers: OK326868- OK326871). The associated BioProject, BioSample numbers, and SRA are PRJNA771243, SAMN22263148-SAMN22263151, and SRR16469678- SRR16469681.

## Abbreviations

AICc_wt: Akaike Information Criterion cumulative weight
BI: Bayesian inference
bp: Base pair
BS: Bootstrap
ESS: Effective sample size
IGSs: Intergenic spacers
IR: Inverted repeat
ITS: Internal transcribed spacer
LDD: Long distance dispersal
LSC: Large single copy
MCMC: Markov chain Monte Carlo
ML: Maximum Likelihood
MRCA: Most recent common ancestor
PCGs: Protein coding genes
Pi: Nucleotide diversity
PP: Posterior probability
rRNA: Ribosomal RNA
SSC: Small single copy
SSR: Simple sequence repeat
tRNA: Transfer RNA
