# Supplementary figures and images for: Plastome phylogenomics and historical biogeography of aquatic plant genus Hydrocharis (Hydrocharitaceae)
Source: BMC Plant Biol. 2022 Mar 8;22:106. doi: 10.1186/s12870-022-03483-2 (PMC8903008; doi:10.1186/s12870-022-03483-2)

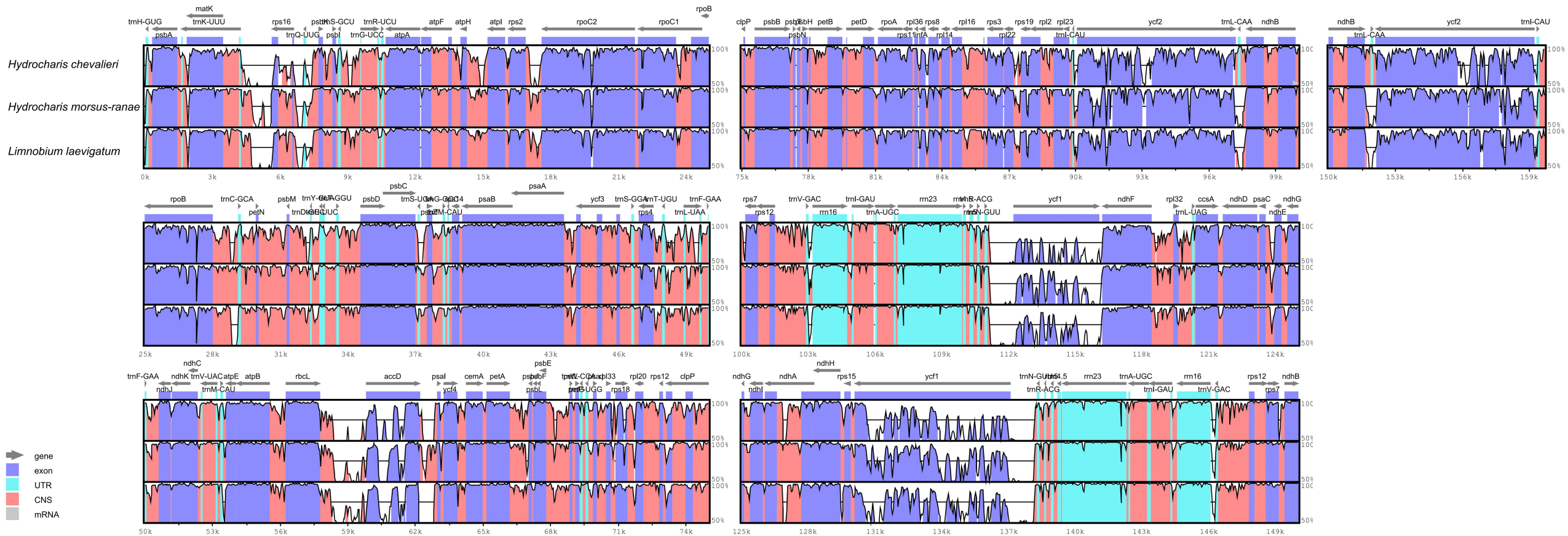

Supplement: Supplementary file 1 — Additional file 1: Figure S1. Genome alignment of plastomes of Hydrocharis species and Limnobium laevigatum. H. dubia was used as a reference. [file 12870_2022_3483_MOESM1_ESM.pdf]

PI

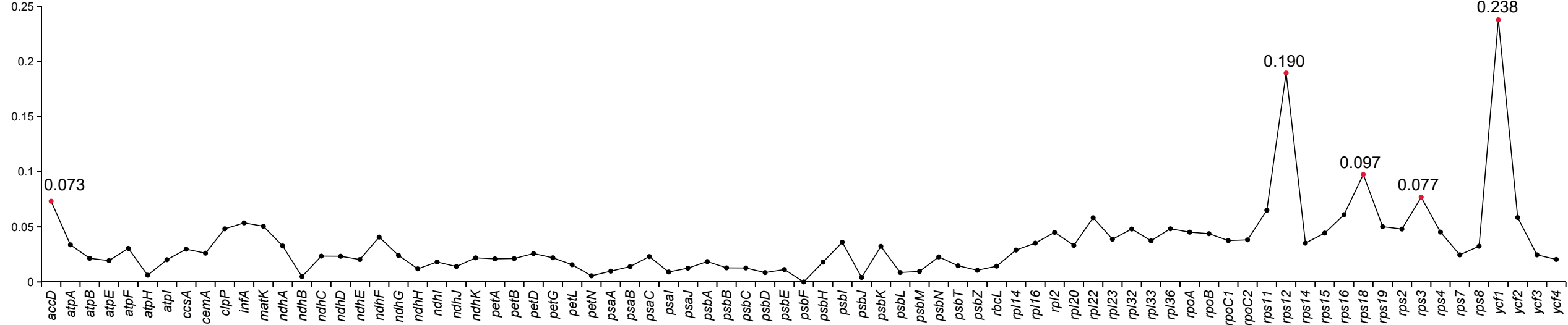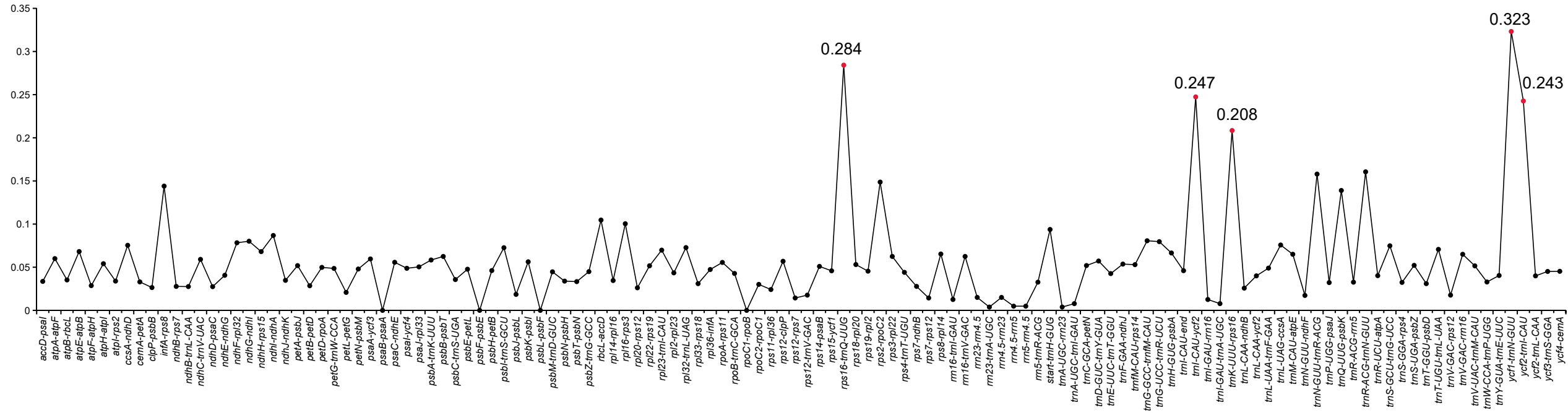

Supplement: Supplementary file 2 — Additional file 2: Figure S2. Nucleotide diversity hotspot regions in plastomes of Hydrocharis species and Limnobium laevigatum. [file 12870_2022_3483_MOESM2_ESM.pdf]

A

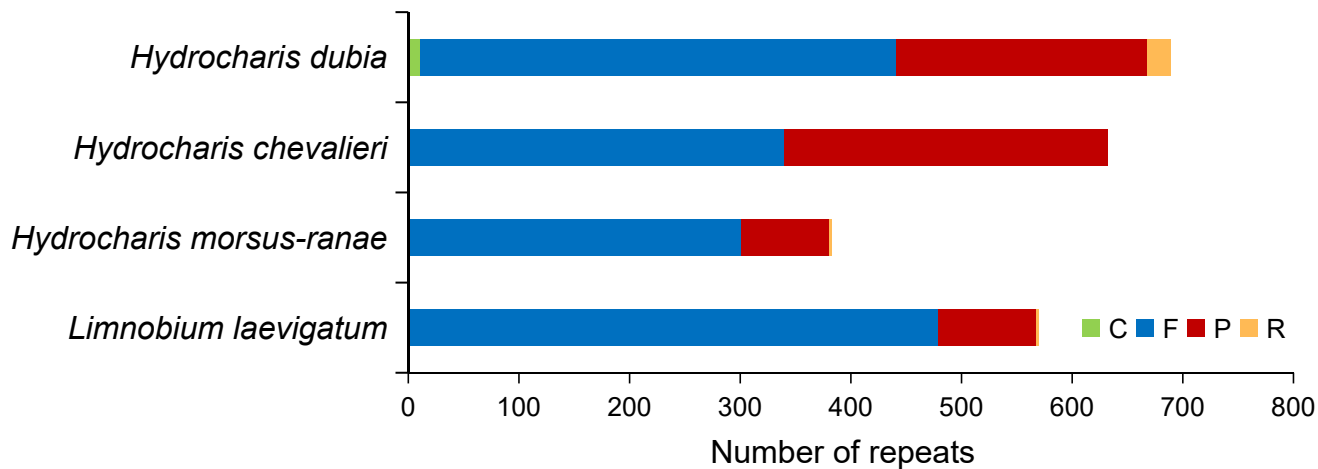

B

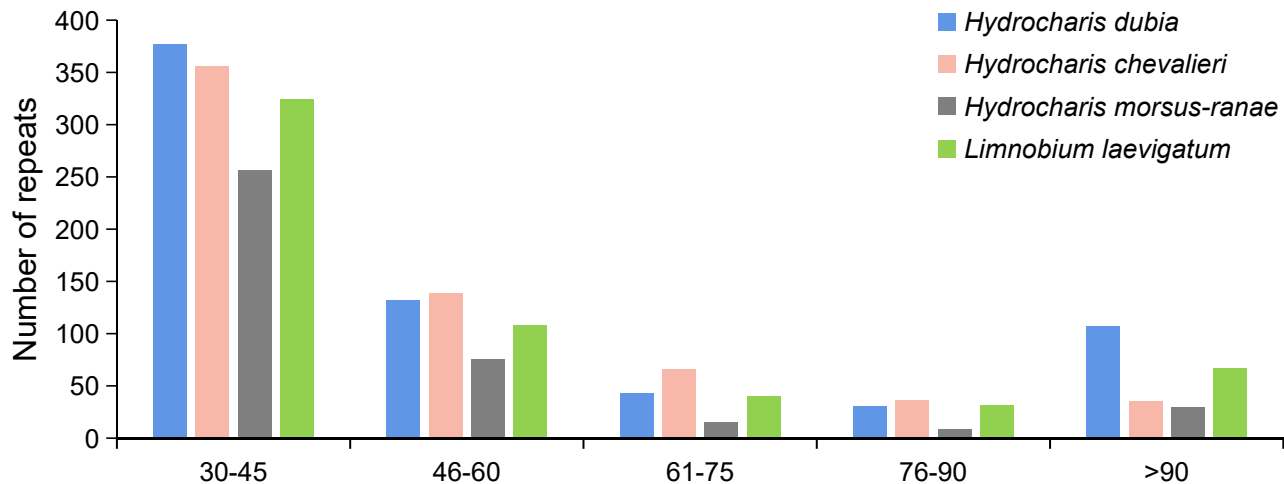

Supplement: Supplementary file 3 — Additional file 3: Figure S3. Analysis of repeat sequences in plastomes of Hydrocharis species and Limnobium laevigatum. (A) Total number of four repeat types. (B) Number of repeats divided by size. [file 12870_2022_3483_MOESM3_ESM.pdf]
