# Supplementary material for: Plastome phylogenomics and historical biogeography of aquatic plant genus Hydrocharis (Hydrocharitaceae)
Source: BMC Plant Biol. 2022 Mar 8;22:106. doi: 10.1186/s12870-022-03483-2 (PMC8903008; doi:10.1186/s12870-022-03483-2)
Supplement: Supplementary file 5 — Additional file 5: Table S2. List of the species downloaded from GenBank and Ross et al. (2016) used for phylogenomic and dating analyses in this study. [file 12870_2022_3483_MOESM5_ESM.docx]

| **Species** | **Family** | **Source** |
| --- | --- | --- |
| *Enhalus acoroides* | Hydrocharitaceae | Ross et al. (2016) |
| *Enhalus acoroides* | Hydrocharitaceae | NC_048519 |
| *Thalassia hemprichii* | Hydrocharitaceae | NC_043774 |
| *Thalassia testudinum* | Hydrocharitaceae | Ross et al. (2016) |
| *Halophila beccarii* | Hydrocharitaceae | NC_051970 |
| *Nechamandra alternifolia* | Hydrocharitaceae | Ross et al. (2016) |
| *Vallisneria americana* | Hydrocharitaceae | Ross et al. (2016) |
| *Najas flexilis* | Hydrocharitaceae | NC_021936 |
| *Najas guadalupensis* | Hydrocharitaceae | Ross et al. (2016) |
| *Limnobium laevigatum* | Hydrocharitaceae | Ross et al. (2016) |
| *Hydrocharis morsus_ranae* | Hydrocharitaceae | Ross et al. (2016) |
| *Elodea canadensis* | Hydrocharitaceae | Ross et al. (2016) |
| *Elodea canadensis* | Hydrocharitaceae | NC_018541 |
| *Egeria najas* | Hydrocharitaceae | Ross et al. (2016) |
| *Apalanthe granatensis* | Hydrocharitaceae | Ross et al. (2016) |
| *Ottelia cordata* | Hydrocharitaceae | NC_046891 |
| *Ottelia ovalifolia* | Hydrocharitaceae | Ross et al. (2016) |
| *Blyxa aubertii* | Hydrocharitaceae | Ross et al. (2016) |
| *Lagarosiphon major* | Hydrocharitaceae | Ross et al. (2016) |
| *Stratiotes aloides* | Hydrocharitaceae | Ross et al. (2016) |
| *Alisma triviale* | Alismataceae | Ross et al. (2016) |
| *Baldellia ranunculoides* | Alismataceae | Ross et al. (2016) |
| *Hydrocleys nymphoides* | Alismataceae | Ross et al. (2016) |
| *Butomus umbellatus* | Butomaceae | Ross et al. (2016) |

**Table S2** List of the species downloaded from GenBank and Ross et al. (2016) used for phylogenomic and dating analyses in this study.
